# Multicomponent positive psychology intervention for health promotion of Brazilian retirees: a quasi-experimental study

**DOI:** 10.1186/s41155-019-0119-2

**Published:** 2019-02-28

**Authors:** Helen Durgante, Débora Dalbosco Dell’Aglio

**Affiliations:** 1 Universidade Federal do Rio grande do Sul – UFRGS, Instituto de Psicologia, Ramiro Barcelos 2600, sala 115, Santa Cecília, Porto Alegre, RS 90035-003 Brazil; 2 Uni LaSalle Canoas, Av. Vítor Barreto, 2288 - Centro, Canoas, RS 92010-000 Brazil

**Keywords:** Positive psychology intervention, Retiree, Health promotion, Program evaluation, Multicomponent

## Abstract

This study evaluated the effects and impact of a multicomponent positive psychology program for health promotion of retirees. A quasi-experimental longitudinal design was used, and the baseline and end of intervention evaluations were analyzed. The intervention consisted of six weekly group sessions for 2 h each. Eighty-eight retirees (females = 72) aged 49–86 (*M =* 65.66, SD *=* 7.53) from South Brazil took part in the study, 54 (females = 48) in the experimental group (*M =* 66.37 years old, SD *=* 7.42), and 34 (females = 24) waitlist controls (*M =* 64.53 years old, SD *=* 7.68). The instruments used were a sociodemographic questionnaire and the Brazilian version of the scales: Interpersonal Reactivity Index; 12-item General Health Questionnaire; Life Orientation Test-Revised;14-item Perceived Stress Scale; Resilience Scale; and Satisfaction with Life Scale. Mixed factorial ANOVA models revealed significant decrease in depression and anxiety symptoms and perceived stress levels, whereas improvement in life satisfaction and resilience was detected in the experimental group at the end of the program. No main effect was found for empathy and optimism. Interaction effects yielded significant difference between groups for measures of empathy, optimism, depression, and anxiety symptoms after the program. There was no significant interaction effect for the other outcome variables. The usefulness and applicability of this model of intervention to aid future health practices and policies are discussed. Contextual issues in the fields of health promotion and disease prevention in Brazil are also problematized.

## Introduction

One of the greatest public health achievements in the last century is that of global population longevity. This entails a process of demographic transition with changes in mortality and fertility rates (United Nations, 2017). Various factors are associated with population aging worldwide such as efficient approaches to reduce infant mortality, improvements in basic living standards and healthcare services, and also the provision of education for health practices and healthy lifestyles (World Health Organization, 2015). As a result of this, relatively new phenomenon of the inverted parameter of the population age pyramid is a rapid increase in the number of retired people and retirement length in an individual’s lifespan (UN, 2017).

The challenges that may come with retirement, however, include socioeconomic changes, loss of social status and personal identity, reduced relationships attained in previous employment, loneliness, higher risk for depression, and health-related outcomes (Barbosa, Monteiro, & Murta, 2016). Additionally, chronic health conditions and multi-morbidities are usually more prevalent as individuals reach advanced ages (WHO, 2015). All these factors put together may pose additional threats to individuals’ health and well-being and also to their families and social relations, once chronic conditions tend to result in negative psychological outcomes and poorer health (Sutipan, Intarakamhang, & Macaskill, 2016).

Nonetheless, given the heterogeneous health functioning as aging takes place, health conditions are highly influenced by individuals’ emotional/psychological wellness and lifestyles. Alternatively, health promotion programs for retirees present the potential to not only improve health determinants by reducing the likelihood of risk factors for disease but also healthcare by decreasing the burden of illness costs to society (Golinowska, Groot, Baji, & Pavlova, 2016). In fact, according to data from a recent review, reduced risk factors for cognitive function decline in older adults tend to be more likely achieved via multicomponent health interventions (Fessel, Mann, Miyawaki, & Rosenberg, 2017). This pattern of intervention usually has ample coverage (hospitals, clinics, or within the health system), as opposed to being delivered at the individual patient level. Additionally, multicomponent designs should combine outcome variables and a variety of methods and activities to achieve desired outcomes; such complex designs should take into account the evaluation of interaction effects between components, across different time sets (Campbel, & Bonell, 2015).

Similarly, the evaluation strategies employed are crucial to estimating expected effects of health interventions (direction and magnitude of changes in outcome variables). Systematic evaluations also allow decisions regarding the continuity, or not, and needs of adaptation according to the intervention’s aims. The evaluation protocol may include: *process evaluation* to assess whether the implementation process is in line with the intervention proposal (procedures, resource allocation, and so on); *effect evaluation* to check the results and scope of changes in expected variables at the end of the intervention; and/or *impact evaluation* via the inclusion of a control group to infer whether changes may be attributed to the intervention (Sarriera, Bedin, Strelhow, & Sarriera, 2018).

In terms of applied psychology for health promotion and disease prevention, theoretical foundations of positive psychology interventions (PPIs) set the basis of an empirically supported approach to building protective factors for health. PPIs’ aims are essentially to foster positive cognitions, feelings, and behaviors throughout the life course (Magyar-Moe, Owens, & Conoley, 2015; Sin & Lyubomirsky, 2009). In other words, PPIs focus on holding a balance between people’s strengths (health buffers from pathology) and weaknesses, as opposed to only focusing on the reduction of mental illnesses, deficits, and what is dysfunctional in one’s life.

Positive intervention designs include planned treatment methods or activities, to enhance individuals’ positive feelings, behaviors, and/or cognition (Sin & Lyubomirsky, 2009). Current evidence suggests group PPIs applied to positive psychotherapy—a particular case of PPIs designed for clinical use—for mild to moderate depression patients throughout the USA, resulted in decreased depressive symptomatology, conversely, increasing life satisfaction, when compared to no-treatment controls (Seligman, Rashid, & Parks, 2006).

Additionally, constructs such as optimism, empathy, and meaning have been associated with improved subjective well-being, health indicators of symptom relief, self-worth, and empowerment of adult patients with psychosis (Riches, Schrank, Rashid, & Slade, 2016). Results from research with elderly individuals have related finding meaning or purpose in life to decreased risks of developing Alzheimer’s disease (Boyle, Buchman, Barnes, & Bennett, 2010), cardiovascular issues (Kim, Sun, Park, & Peterson, 2013), and mortality (Hill & Turiano, 2014). Nevertheless, available data illustrate that the purpose in life/meaning tends to decline with age, representing a risk factor itself for the physical and mental health of the elderly (Ryff, 2014).

Regarding other strengths found as protective factors for health, a Spanish positive intervention delivered to 46 elderly, aiming at developing gratitude and forgiveness, revealed significantly decreased rates of state anxiety and depression, while improvements in memory, life satisfaction, and subjective well-being were detected (Ramírez, Ortega, Chamorro, & Colmenero, 2014). Likewise, it was evidenced increased rates of happiness, decreased levels of negative concern, and systolic blood pressure after 67 Spanish adults took part in a group intervention for the development of strengths, positive emotions, and emotional regulation (Jiménez, Izal, & Montorio, 2016).

Despite evidence indicates that PPIs have a significant impact on a variety of health indicators, recent meta-analyses showed that the number of interventions designed and delivered specifically to elderly and/or retired populations is, yet, way below the ideal level. That is, when compared to other population strata, only four interventions out of 51 (Sin & Lyubomirsky, 2009), and seven out of 39 studies compiled (Bolier et al., 2013) have been delivered to people aged 60 years or above. Sutipan et al. (2016) conducted an extensive review to evaluate the effects of PPIs on levels of well-being of healthy elderly. What they found was only eight studies met the inclusion criteria presenting consistent results of improvement in well-being and alleviation of depressive symptoms. The review also identified long-term effects, particularly enduring for gratitude interventions.

In the Latin American context, however, the methodological quality of PPIs was identified as questionable in a previous review (Durgante, 2017). Lack of comparison groups, baseline, post-test and follow-up assessments, provision of manualized guides for replication studies, and having the different personnel to implement and evaluate outcomes were all on a regular basis in the designs evaluated. In Brazil, the reality in the number of PPIs applied for health promotion targeting elderly/retirees is even more sparse, and methodological quality remains unsatisfactory, with very few controlled trials, lack of methodological rigor of program evaluation—qualitative or quantitative methods, also interventions’ main goals are still on disease prevention and rehabilitation practices (Machado, Gurgel, & Reppold, 2017; Reppold, Gurgel, & Schiavon, 2015). Hence, the need to design, implement, and evaluate the results of PPIs for elderly/retirees becomes a major issue to be addressed in Brazil, to assess the effects/impact of these interventions on this growing population.

Bearing this in mind, we carried out a previous feasibility study with *Programa Vem Ser:* a multicomponent positive psychology program for health promotion of retirees. Different feasibility criteria were assessed to identify and rectify possible faults in the design and implementation process of the intervention (Durgante, Navarini, & Dell'Aglio, in press). Preliminary results of the program were satisfactory, allowing to make changes in the program structure (e.g., change in the average duration of all sessions of the program, from 1 h 30 min to 2 h each; duration of the admission interview from 30 min to approximately 40 min–1 h) and implementation methods (relocation of observers in the room, etc.).

The present study, thus, will add to the knowledge of PPIs’ effects on health promotion of retirees/elderly, as well as extend previous findings from *Programa Vem Ser*, at this time, including results from six experimental groups as efficacy trials of the program. The current study aims were to evaluate the effects and impact of *Programa Vem Ser*: a multicomponent positive psychology program for health promotion of retirees. The hypothesis predicted that participants in the intervention group would present significant improvement in measures of empathy, general health, life satisfaction, perceived stress, resilience, and optimism by the end of the intervention. Additionally, the experimental group is hypothesized to present increased scores on all outcome measures relative to no-treatment controls. Different efficacy criteria were employed and analyzed in accordance with treatment guidelines for the best available practices in the field (Appelbaum et al., 2018).

### Intervention design: *Programa Vem Ser*

*Programa Vem Ser* is a health promotion program to provide tools—education for health—so individuals may identify and/or develop strengths to best manage and effectively cope with life stressors/demands, thus building on resilience. The program was based on empirical perspectives from cognitive behavioral therapy (CBT) for the treatment and alleviation of psychopathology, also on positive psychology interventions (PPIs) to improve general health and well-being (Bolier et al., 2013; Knapp & Beck, 2008; Seligman, Steen, Park, & Peterson, 2005; Sin & Lyubomirsky, 2009; Sutipan et al., 2016). The program consists of a pre-test assessment, six weekly sessions (2 h each for 5 > 15 participants), followed by a post-test evaluation of primary outcome measures. The program is designed to be implemented by a trained moderator and two trained observers in all sessions.

The strengths worked in each session were selected according to literature data which indicate fundamental gains for the maintenance of health and disease prevention in different elderly samples worldwide after improving/developing such strengths. An additional point for including some strengths in detriment of others is that not enough programs have been conducted to promote such strength in Brazil for this target population. Similarly, specificities of the Brazilian sample were taken into account such as the socioeconomic barrier to getting to health services, limited health promotion resources available for this age group, and little evidence of the effects of such intervention format in this given context (Durgante, 2017; Reppold et al., 2015; Seibel, DeSousa, & Koller, 2015). In the case of adapting to the Brazilian context, strengths should be considered as a unidimensional measure, as opposed to classified according to different categories of virtues, which seems to be a trend in international studies. Themes/strengths, learning objectives, and activities worked in each session of the program are available in the Appendix.

Perspectives from PPIs, also CBT techniques, were adapted in this intervention program for the Brazilian retiree. The scientific criteria for good practice in efficacy trials (Appelbaum et al., 2018) used in the design of the program included: literature search and systematic reviews (Durgante, 2017); expert consultations to design and adapt the intervention to cultural specificities of the target population; members of staff training including getting familiar with background literature, program objectives, and implementation process protocol; internal pilot study with the program delivered to experts (*N* = 10) to allow changes in the design and implementation procedures; and a feasibility study for the target population (*N* = 11).

## Methods

### Design

This study utilized a quasi-experimental longitudinal design with an experimental group (EG) (*Programa Vem Ser*) and a non-equivalent control group (CG ) ([technical term] no-treatment waiting list). Randomization was not considered feasible due to contextual issues in Brazil and thus not applied in the current study. In other words, the choice for a quasi-experimental design helped to avoid long waitlists, which is a recurrent reality in the Brazilian public health settings, preventing individuals to receiving care when necessary. Ethical concerns that assure public health services are promptly available to participants soon after baseline assessment was also considered imperative for the decision to use a quasi-experimental design. Outcome variables were assessed at baseline (pre-intervention; [T1]), and post-test (immediately after intervention [T2]). Evaluations were carried out at the same times for both groups (EG and CG) by means of self-reported measurements. Multiple sites for data collection included six collection points at T1 and T2 conducted along the year of 2017 for the experimental group and controls, simultaneously. The independent variable (IV) was the intervention program (*Programa Vem Ser*), and the dependent variables (DVs) were empathy, general health, life satisfaction, perceived stress, resilience, and optimism. The Ethics Committee of Universidade Federal do Rio Grande do Sul (UFRGS, Brazil) approved the study protocol.

### Participants

Participants were recruited from the municipal community and health centers, retirees associations, and groups for older adults in the Metropolitan Region of Porto Alegre, RS, Brazil. The advertising also included posts in social media/network, in a local newspaper, on the University webpage, and radio. A total of 105 retirees from South Brazil (females = 85, males = 20) aged 49–86 (*M =* 65.84, SD *=* 7.28*)* were recruited as a voluntary opportunity sample. Six participants completed a pre-test evaluation (T1) but did not start the intervention (females = 4, males = 2) aged 53–69 (*M =* 63, *SD =* 6.22). Overall, 99 participants (females = 81, males = 18) aged 49–86 (*M =* 66.01*, SD =* 7.33) took part in the program. Of those, 34 people (females = 24, males = 10), whose ages ranged from 50 to 83 years (*M =* 64.53, *SD =* 7.68) were allocated to the CG, and 65 people (females = 57, males = 8) age range between 49 and 86 (*M =* 66.78 years old, *SD =* 7.08) participated in the EG. Allocation of conditions was according to participants’ self-selection to take part in the intervention group or only fill in the evaluation protocol to receive feedback on their health measures assessed (CG). The inclusion criteria provided in the advertisements were (a) be retired, (b) be literate, and (c) complete the evaluation protocol.

For the post-test analysis, 11 participants failed to complete the health evaluation protocol (T2) due to the following reasons: eight dropped out during the sessions of the program (health-related issues [2], personal reasons [1], wanted to be in groups with more male participants [1], had to look after grandchildren [2], did not feel comfortable in a group [1], did not specify [1]) and three participants finished all sessions of the program but, however, could not stay to complete post-test assessment (bereavement [1], had to look after spouse [1], had to leave early [1]). Only the data from those who concluded T2 evaluation (*n* = 88; EG = 54, CG = 34; female = 72; aged 49–86 years [*M =* 65.66*, SD =* 7.53]) were included in the analysis. The T2 attrition rate for this study (16.9%) was lower than that found (37.7%) in a similar national longitudinal study about positive psychology (Damásio, Golart, & Koller, 2015) and in a meta-analysis (42%) of longitudinal studies internationally (Roberts & DelVecchio, 2000).

### Instruments

All participants answered an admission questionnaire during a semi-structured interview. Questions included sociodemographic information (age, educational background, marital status, family configuration, number of residents in the house, previous work position, time of service, time and type/classification of retirement), post-retirement work (paid or voluntary), if respondents look after somebody (grand/children, parents, spouse, relatives, etc.), were/are in psychological treatment, have received psychiatric diagnosis, perceived social support, and current health conditions. The protocol was based on recent data on retirement adjustment predictors (Barbosa et al., 2016).

#### The following instruments were used for the evaluation of efficacy criteria

Interpersonal Reactivity Index, IRI **(**Davis, 1983; Brazilian version adapted by Sampaio, Guimarães, Camino, Formiga, & Menezes, 2011). The Brazilian version is a 26-item on a 5-point Likert scale to evaluate cognitive (perspective taking; fantasy) and affective (empathic concern; personal distress) aspects of empathy. The adaptation study of the scale presented satisfactory internal consistency = 0.86 (Sampaio et al., 2011) and αs 0.77 and 0.85 at T1 and T2 in this study.

12-item General Health Questionnaire (GHQ-12) (Goldberg & Williams, 1988; Brazilian version adapted by Pasquali, Gouveia, Andriola, Miranda, & Ramos, 1994). This measure assesses general (non-psychotic) psychiatric morbidity (anxiety and depression symptomatology), based on a four-point scale of participants’ perceived health in the last few weeks. It has been used for the health screening of general and clinical adults worldwide. The higher the scores obtained, the more anxiety and depression symptoms. The Brazilian version of the scale showed good internal consistency (*α* = 0.80), and in this study, *α* at T1 was 0.88 and T2 0.89.

Life Orientation Test-Revised (LOT-R) (Scheier, Carver, & Bridges, 1994; Brazilian version adapted by Bastianello, Pacico, & Hutz, 2014). This 10-item (4 fillers; 3 to measure optimism; 3 to measure pessimism) 5-point Likert scale examines optimism in a unidimensional measure. High scores indicate higher levels of optimism. In the validation study, it presented high internal consistency (*α* = 0.80), and in this study, Cronbach alphas ranged from 0.63 to 0.62.

Perceived Stress Scale (PSS-14) (Cohen, Kamarck, & Mermelstein, 1983; Brazilian version adapted by Luft, Sanches, Mazo, & Andrade, 2007). This is a 14-item 5-point Likert scale about respondents’ appraisal of life situations as overloading, unpredictable, and uncontrollable, over the past month. Studies with the Brazilian version of the scale indicated high internal consistency (*α* = 0.82) and construct validity (Luft et al., 2007), as well as similar psychometric properties to the original version of the scale, and precision to detect intragroup differences (Faro, 2015). The Cronbach alphas at T1 was 0.72, and at T2 0.73.

Resilience Scale (RE) (Wagnild & Young, 1993; Brazilian version adapted by Pesce et al., 2005). This is a 25-item 7-point Likert scale to measure respondents’ positive psychosocial adaptation to extremely stressful/demanding life events. Cross-cultural adaptation study to Brazilian Portuguese has shown semantic equivalence, construct validity, internal consistency (*α* = 0.80). Scores vary from 25 to 175 points, while higher scores indicate higher resilience. Internal consistency was 0.91 and 0.86 at T1 and T2, respectively.

Satisfaction with Life Scale (SWLS) **(**Diener, Emmons, Larsen, & Griffin, 1985; Brazilian version adapted by Zanon, Bardagi, Layous, & Hutz, 2013). This 5-item Likert scale evaluates satisfaction with life as the cognitive component of subjective well-being. Previous studies with the Brazilian version of the scale have shown adequate psychometric properties and satisfactory internal consistency (*α* = 0.87) (Zanon et al., 2013). *α*s were 0.86 at T1 and T2 in this study.

### Procedures

Participants who met the inclusion criteria self-selected to the EG or the CG. The EG was subjected to an interview (from 40 min to 1 h) and carried out in the University research group office. Immediately after the admission interview, participants completed the evaluation protocol as a baseline assessment (T1). The evaluation protocol was administered by a different researcher to avoid contamination of data, and the measurements were counterbalanced to reduce order effect (Hair, Black, Babin, & Anderson, 2013). For the CG, those who were not able to complete the protocol face-to-face were sent it via e-mail or by post (4A paper). All participants completed the information sheet (informed consent form) explaining the purpose of the study, risks and benefits of participation, and received the researcher’s and the Ethics Committee’s contact details for any clarification. All sessions of the program were carried out in a university classroom. The EG was re-evaluated at the end of the last session of the program (T2), while contact was made to controls for re-evaluations at the same period. Participants were provided with feedback on their health indicators after evaluations. The CG was offered the possibility to participate in subsequent intervention groups. The intervention was delivered by a trained psychologist.

### Data analysis procedures

Prior analysis was conducted to screen for normality (Shapiro-Wilk test) and outliers at baseline assessment of group differences in sociodemographic status and for each of the DVs. All statistical analyses were performed using the IBM Statistical Package for the Social Sciences (SPSS Statistics, Version 21). Homogeneity of variance between groups was checked with Levene’s test (*α* > 0.05) and variance-covariance matrices with Box’s *M* test (*α* > 0.001). Descriptive and inferential statistics were conducted for sociodemographic data. Chi-square tests were used for categorical data and independent-sample *t* tests for continuous data. Reliability coefficients (Cronbach’s alpha) were calculated for the measurements utilized with the current sample. To analyze the intervention effects (within-group differences at T1–T2) and impact (between-group differences at T2), data were assessed using models of mixed-design analysis of variance (split-plot ANOVA) with between-subject factor as the treatment, having two levels (CG and EG), and within-subject factor as the time of assessment, also with two levels (T1 and T2). ANOVA results were checked for pre-test (T1)–post-test (T2) changes in the outcome measures within groups (main effects). Within-subjects’ *t* tests (available in Table 2) were carried out to further investigate whether main effects at T1 and T2 for the EG alone (split file) were statistically significant—after isolating the effects from the CG. Effect size for *t* tests (*d*) was based on the following classification: 0.20–0.49 = small; 0.50–0.79 = moderate; above 0.80 = large (Field, 2013).

Interaction effects from the ANOVA models were analyzed for between-group differences (EG versus CG) at T2. ANOVA effect size was based on partial eta squared (ηp^2^) according to the following values: smaller than 0.01–0.05 = small; 0.06–0.13 = medium; above 0.14 = large (Field, 2013).

## Results

### Baseline analysis

The overall data presented normal distribution (non-significant results in Shapiro-Wilk test statistics), homogeneity of variance between groups (Levene’s test > 0.05) and variance-covariance matrices (Box’s *M* test > 0.001), no significant outliers, and adequate sample size (*n* > 30 in each group), despite the difference in the number of subjects in each condition (Hair et al., 2013). Results from chi-square test indicated that there was no statistically significant difference (*p* > .05) between groups (CG vs EG) for gender, educational background, retirement type, participation in any sort of preparing for retirement programs (PRPs), post-retirement work (paid or voluntary), current health statues, if participants were carers of somebody, perceived social support, and diagnostic of psychopathology. However, a higher percentage of individuals reported to have a partner in the CG (67.6%) when compared to those in the experimental condition (39.4%), and this difference was significant (*p* = .012). Conversely, a significantly higher percentage of individuals reported to live alone in the EG (43.7%) compared to controls (11.8%) (*p* = .003).

Two-tailed independent sample *t* tests showed the groups did not significantly differ on age, number of residents in the house, number of children, service time, and retirement time. No statistically significant difference was found between groups for any of the DVs at baseline. The means, standard deviations, degrees of freedom (df) percentages (%), chi-square (*X*^2^) and independent sample *t* test statistics results, and *p* values for each outcome measure in both groups are presented in Table 1.

**Table 1:** Descriptive and inferential statistics of data for the CG (*n* = 34) and EG (*n* = 54)

		CG (%)	EG (%)	*X* ^2^	df	*p*
Gender	Female	70.6	88.9	3.54	1	.06
	Male	29.4	11.1			
Educational level	Up to high school	52.9	42.6	0.530	1	.467
	Above high school	47.1	57.4			
Retirement type	By age	23.5	9.3	3.42	2	.18
	Time of service	55.9	68.5			
	Especial	20.6	22.2			
PRPs	Yes	2.9	16.7	2.659	1	.103
	No	97.1	83.3			
Post-retirement work	Yes	44.1	50	0.102	1	.750
	No	55.9	50			
Health statues	Positive	85.3	68.5	2.301	1	.129
	Negative	14.7	31.5			
Carer	Yes	32.4	51.9	2.473	1	.116
	No	67.6	48.1			
Social support	Yes	97.1	98.1	0.001	1	1.00
	No	2.9	1.9			
Psychopathology	Yes	20.6	27.8	0.256	1	.613
	No	79.4	72.2			
Partner	Yes	70.6	40.7	6.302	1	.012*
	No	29.4	59.3			
Life alone	Yes	11.8	40.7	7.081	1	.008*
	No	88.2	59.3			
	CG: *M* (SD)	EG: *M* (SD)	*t*	df	*p*	CI (95%)
Age	64.53 (7.68)	66.37(7.42)	− 1.11	86	.267	(− 5.11–1.43)
Service time	24.67 (9.68)	28.92 (10.03)	− 1.96	86	.053	(− 8.55–0.06)
Retirement time	12.40 (10.17)	13.91 (11.72)	− 0.620	86	.537	(− 6.36–3.34)
Perceived stress	23.03 (6.67)	22.43 (7.26)	0.391	86	.696	(− 2.46–3.66)
Empathy	93.68 (11.53)	94.31 (10.05)	− 0.274	86	.785	(− 5.27–3.99)
General health	23.18 (5.30)	23.41 (6.49)	− 0.174	86	.862	(− 2.87–2.40)
Optimism	24.26 (3.51)	24.41 (3.84)	− 0.175	86	.861	(− 1.76–1.47)
Resilience	139.38 (13.88)	138.43 (21.95)	0.227	86	.821	(− 7.42–9.33)
Life satisfaction	26.47 (6.68)	26.39 (5.68)	0.061	86	.951	(− 2.56–2.73)

Note: *CG* control group, *EG* experimental group, *df* degrees of freedom, *M* mean, *SD* standard deviation, *CI* confidence interval, *PRPs* preparing for retirement programs

*p < .05

### Program effects and impact evaluation

By analyzing each measure across time point results indicated statistically significant main effect of time of measurement on general health (F(1,86) = 8.97, *p =* .004, ηp^2^ *=* 0.094), life satisfaction (F(1,86) = 3.81, *p =* .05, ηp^2^ *=* 0.043), perceived stress (F(1,86) = 20.26, *p =* .001, ηp^2^ *=* 0.19), and resilience (F(1,86) = 6.26, *p =* .014, ηp^2^ *=* 0.07). There was no significant main effect of time on empathy (F(1,86) = .098, *p =* .755) and optimism (F(1,86) = 5.49, *p =* .314).

Results from subsequent within-subjects *t* tests (split file) indicated improvement in general health (*p = .*001; CI *=* 2.462–6.279), life satisfaction (*p = .*002; CI *=* − 2.572 to – 0.613), perceived stress level (*p = .*002; CI *=* 1.377–5.586), and resilience (*p = .*011; CI = − 12.850 to –1.706) in the EG. Although within-subjects *t* test revealed a statistically significant increase in optimism for the EG from baseline to post-test (*p =* .007, CI = − 1.993 to – 0.341), for the purpose of this study once no main effect of time was found in the general model (*F* statistics is considered more robust to avoid type I error due to taking systematic error variance between and within groups into account), within group difference in optimism will not be considered significant (Hair et al., 2013). Empathy scores for the EG were not significantly different (*p =* .158; CI *= −* 5.020–0.835) after the completion of the program. There were no statistically significant difference in scores between T1 and T2 for the control group, except from perceived stress levels (*p =* .003, CI *=* 1.416–6.408). The means, standard deviations, test statistics, degrees of freedom (df), *p* values, and effect sizes for each outcome variable across measures (T1 and T2) for the EG, and means and standard deviations for the CG, are available in Table 2.

**Table 2:** Test statistics results for each outcome measure in the EG (*n* = 54) and CG (*n* = 34)

Variables	GE T1: *M* (SD)	GE T2: *M* (SD)	*t*	df	*p*	*d*	CG T1*: M* (SD)	CG T2: *M* (SD)
Empathy	94.31 (10.05)	96.41 (12.90)	− 1.434	53	.158	0.20	93.68 (11.53)	90.85 (12.46)
General health	23.41 (6.49)	19.04 (4.98)	4.594	53	.001*	0.75	23.18 (5.30)	23.53 (5.86)
Life satisfaction	26.39 (5.68)	27.98 (5.51)	− 0.3260	53	.002*	0.28	26.47 (6.68)	27.12 (5.12)
Perceived stress	22.43 (7.26)	18.94 (6.38)	− 3.318	53	.002*	0.51	23.03 (6.67)	19.12 (5.52)
Resilience	138.43 (21.95)	145.70 (13.61)	− 2.620	53	.011*	0.39	139.38 (13.88)	141.91 (11.54)
Optimism	24.41 (3.84)	25.57 (3.57)	− 2.833	53	.007*	0.31	24.26 (3.51)	23.82 (3.36)

*p < .05; *d =* effect size

There were significant interaction effects of time (T1, T2) × group (CG, EG) on general health (*F*(1,86) = 12.39, *p =* .001, ηp^2^ *=* 0.126), empathy (*F*(1,86) = 4.42, *p =* .038, ηp^2^ *=* 0.05), and optimism (*F*(1,86) = 5.03, *p =* .027, ηp^2^ *=* 0.055). These indicate the EG mean scores for those variables significantly improved when compared to waitlist controls at T2. The differences in mean life satisfaction (*F*(1,86) = 0.68, *p* = .412), perceive stress (*F*(1,86) = 0.07, *p = .*794), and resilience scores (*F*(1,86) = 1.46, *p* = .229) between groups at post-test did not reach statistical significance.

### Dropout analysis

Comparisons were conducted between participants who did not complete the program—dropouts—(*n* = 17 from the EG, female = 13; *n =* 6 did not start the program, *n =* 11 did not complete all the sessions, or T2 assessment), aged 53–76 years (*M =* 66.76, SD *=* 5.93), and completers (*n =* 88, CG = 34, EG = 54, female = 72) aged 49–86 years (*M =* 65.66, SD *=* 7.53) with regard to participants’ baseline characteristics.

Two-tailed independent sample *t* tests showed that the groups did not significantly differ on age, number of residents in the house, number of children, service time, retirement time, perceived stress, resilience, empathy, and optimism scores (*p* > .05). Statistically significant difference was found between groups for depression and anxiety symptoms (*p =* 0.05), dropouts presenting higher scores at baseline (*M =* 26.82, SD = 9.67), when compared to completers (*M =* 23.32, SD = 6.03). Chi-square results indicated no statistically significant difference (*p* > .05) between groups for gender, educational background, marital status, retirement type, participation in PRPs, post-retirement work (paid or voluntary), current health status, if participants were carers of somebody, and diagnostic of psychopathology. However, significantly higher percentage of individuals (97.7%) reported to have perceived social support in the completers’ group (*p* = .004).

## Discussion

*Programa Vem Ser* was efficacious in improving health indicators in this Brazilian retiree sample. As for the main effects detected—which significantly improved over the course of the program from baseline to T2 in the EG—small effects were detected for life satisfaction (*d =* 0.28) and resilience (*d =* 0.39), whereas medium effect sizes were found for perceived stress (*d =* 0.51), and general health results (*d =* 0.75)—depression and anxiety symptoms. Contrary to expectations, results presented no statistically significant main effect of the program on empathy and optimism. Additionally, despite the low effect size (around 5% of the variation in error scores), there were significant interaction effects between time of evaluation and group. This was accounted for by the EG presenting higher scores on empathy and optimism in contrast to results of the CG at the end of the intervention. Interaction effects also indicated 12.6% of the variation in error scores of general health and were attributed to the intervention—medium effect size. The other variables did not significantly improve in contrast with the controls’ at T2.

One important point to consider when using effect size as a stand-alone estimate to determine the clinical relevance of the findings (especially in cases of preliminary efficacy trials) is that categorizations (small-medium-large effects) are based on group mean differences instead of on clinical criteria/thresholds. In this case, a small-to-medium effect size is expected to be achieved in the majority of preliminary treatment trials due to sample-size restraint. This was identified in two previous meta-analyses which the purposes were to analyze the effects of PPIs on the well-being and depression of mainly non-clinical samples (Bolier et al., 2013; Sin & Lyubomirsky, 2009). According to Sin and Lyubomirsky (2009), large effects of PPIs were detected on well-being (*r* = 0.29) and depression (*r* = 0.31), whereas Bolier et al. (2013) found smaller effects on the same variable (well-being *d =* 0.34; depression *d =* 0.23).

Similarly, a more recent meta-analysis evaluated whether results from PPIs would show similar effects on clinical samples. The findings indicated small effect sizes for well-being (Hedges’ *g* = 0.24) and depression (*g* = 0.23), while moderate effect was found for anxiety (*g* = 0.36) (Chakhssi, Kraiss, Sommers-Spijkerman, & Bohlmeijer, 2018). Taken together, these findings suggest that a small-to-medium effect size may serve to signal the magnitude of change detected. What they do not indicate, however, is that small effects are less important for the clinical significance and utility of the results in practical terms. In fact, results need not necessarily be statistically significant to be clinically relevant (Aarts, van den Akker, & Winkens, 2014).

Therefore, the effects found in the present study, despite being predominantly categorized as small-to-medium effect sizes, have to be investigated in future effectiveness trials of the program. Subsequent analyses should include, for instance, cumulative percent of participants that reach certain thresholds/cutoff points for particular conditions (clinical or sub-clinical). Effectiveness trials with the program will allow the amplification of the current sample, more ascertained inferences when based on effect-sizes, interpretations of how the findings may be applicable for true effect in the population, and generalization of these results beyond the current sample.

At present, the findings are, nonetheless, consistent with a large body of international research which points out the benefits of PPIs for the physical and mental health of elderly populations (Sutipan et al., 2016). This is a critical issue once cognitive and physical implications, psychological distress, loss of interest, independence, and autonomy in everyday activities may all take place with the progression of age (Barbosa et al., 2016). All these factors increase the likelihood of illnesses, particularly for those individuals who did not plan/prepare for retirement along the life course. In Brazil, a specific legislation that recommends the implementation of retirement preparation programs by the organizations dates back to 2003 (Law no. 10.741, 2003). However, to date, only about 23% of the Brazilian organizations offer such programs to encourage planning for retirement, awareness of health maintenance, and well-being after retirement (Pazzim & Marin, 2016). This reiterates that investments in health promotion practices are urgent in this context, via evidence-based approaches for favorable outcomes in a country where prevention (at last health promotion) is still an exception to the rule.

Another point worth noting regards the structure of *Programa Vem Ser.* In this program, different themes (strengths) were introduced/worked in an attempt to improve participants’ health-related outcomes. Following a single direct-effect rationale, only variables worked along the sessions should present improvement post-intervention. Nonetheless, health as a complex phenomenon and multidimensional construct results from multiple interacting factors. This is why multicomponent interventions are claimed to present advantages when compared to single component ones, especially for primary care services such as health promotion and/or disease prevention (Martín Cantera et al., 2015).

Some components introduced in *Programa Vem Ser* were raising awareness on the role of strengths as protective factors for health (Psychoeducation), being cognitive and behavioral-change oriented, task-directed, and motivation-driven at the individual and social level (establishing and maintaining constructive social relations/environment, leisure, meaningful activities). The results at T2 suggest the integrative/cumulative effects of strengths on various health domains. This is in line with Sin and Lyubomirsky (2009) “shotgun approach” which states that the introduction of different intervention patterns into one was found beneficial for health gains across different age groups. Similar interventions have demonstrated that integrating components into the intervention designs may lead to increased levels of well-being and cognitive functioning in elderly samples (Ramírez et al., 2014). In Brazil, the present results are promising and of great importance, once previous findings have shown, and health conditions and social support are significant predictors of happiness in retirees from urban and rural areas (Amorim, França, & Valentini, 2017).

It is worth noting that overall, 25% of the sample in this study (CG = 20%; EG = 28%) reported being experiencing some sort of psychopathology—sleep disturbance, mild/moderate depression, and/or anxiety. In terms of depression symptoms, in particular, a 30-year follow-up longitudinal study revealed depressive episodes which were the main factor associated with life satisfaction scores amongst retirees (Wilhelm, Geerligs, & Peisah, 2013). Similarly, a handful of studies suggest psychological health appears as the most significant predictor of prospective life adjustment as retirement and aging take place (Bretherton & McLean, 2016; Steptoe, Deaton, & Stone, 2015). Thus, having achieved a reduction of depression and anxiety symptoms and perceived stress levels, improvements in life satisfaction and resilience in the intervention group demonstrate the critical role that *Programa Vem Ser* may play in developing protective factors against health impairments. This program in the format of a PPI may be beneficial for not only health promotion but also for an applied preventive science for this age group in the current context.

Similarly, nearly 60% of participants in the EG did not have a partner, and more than 40% reported to live alone. Previous data suggest that having a partner is considered a key predictor of a better retirement adjustment (Barbosa et al., 2016), and also, perceived care from a partner accounts for higher rates of life satisfaction of retirees (Wilhelm et al., 2013). Interestingly, life satisfaction was introduced as a fundamental variable in the latest broader framework for successful aging, while factors that lead to life satisfaction are weighted differently across cultures (Jensen, Dungan, Goates, & Thacker, 2017). By examining the profile of the participants of *Programa Vem Ser*—living alone and not having a partner—it is feasible to argue that these individuals were a higher risk group. In this case, a minor increase in health indicators may represent great gains for a more successful aging process (Alves, Maia, & Nardi, 2014). Despite some results were statistically non-significant when compared with controls’, this does not mean health improvements achieved are less valuable. Even no significant findings may be relevant for clinical practices to add up knowledge and broaden the understanding of the influencing factors on successful aging (Jensen et al., 2017).

As to interaction effects, the obtained results indicated the impact of the program on measures of general health, optimism, and empathy. This was due to, on the one hand, decreased levels of optimism and empathy and increased scores of depression and anxiety symptoms in the CG, and on the other hand, improvement in optimism and empathy, and general health scores decline in the EG. The results at T2 suggest that the program may lessen the effects of time on health indicators and be prophylactic to the worsening of optimism, empathy, and general health scores. In fact, according to data from the Gallup World Poll, an over 160-country ongoing survey on well-being (Steptoe et al., 2015), Latin American samples tend to present falling well-being rates with age. Hence, it is imperative to systematize innovative practices to ameliorate health determinants and aid future policies associated with greater health outcomes in this given context.

With regard to empathy, results were rather contradictory, while the main effect was not significant and a statistically significant interaction effect was detected. Further analysis of time by group interaction indicated the scores of the EG significantly increased in contrast with empathy scores decline of the CG at T2. A possible reason for the failure to obtain the main effect on empathy is that only one session of the program focused on this strength specifically. In fact, studies have revealed heritable estimates for affective components of empathy (Hastings, Miller, Kahle, & Zahn-Waxler, 2013; Melchers, Montag, Reuter, Spinath, & Hahn, 2016). This means imprinted, therefore, persistent patterns of behaviors (trait-related) more rigidly integrated into one’s personality.

Personality-related factors pose a challenge to brief-format intervention designs once changes could be expected in the long run. Thus, participants may need a longer period to assimilate/elaborate contents of psychosocial interventions and reformulate knowledge as behavior changes. This process depends on one’s social environment stimuli and brain capacity of neural plasticity to develop new connections and learning (Conway & Slavich, 2017; Sharpe, Martin, & Roth, 2011). As a suggestion for future intervention designs, empathy topics should be made available along, if not in all, at least during different sessions of the program to allow the progressive consolidation of knowledge and the detection of changes in subsequent evaluations more thoroughly.

In terms of optimism, how to address traits in psychosocial interventions so expected changes may be detected is a question that requires considerate care from health professionals. Twin/adoption studies have already revealed moderate heritability estimates for optimism, with random variations due to environmental factors that mediate and influence the expression of optimistic styles (Bates, 2015; Caprara et al., 2009). Because research has shown optimism has been linked to personality factors, it makes sense to question whether optimism-focused interventions should present longer duration. Such interventions could benefit from targeting specific personality traits along the modules, once traits may function as precursors of optimism (Sharpe et al., 2011; Zuckerman, 2003). To try and achieve higher main effects in future interventions, the additional focus could be on personal resources and competence as protective factors over and above optimistic explanatory styles (e.g., hope, self-regulation, positive emotions, social support, etc.). This could help down-regulate pathology development, particularly in cases of cumulative risk exposure such as those encountered in advanced ages.

An additional existing challenge for the Brazilian applied research is the limited number of measures developed or validated specifically on the elderly/retiree populations. This is a common issue encountered in non-English speaking countries, where measures are usually developed or adapted/validated on student samples. Nonetheless, despite the advantage of being a more easy-to-reach sample, students do not represent the general population in many ways. As a result, measures tend to be applied to populations with diverse backgrounds or to samples other than those within which measures were developed in the first place, which decreases the sensitivity to change of the measures (Fitzpatrick, Ziebland, Jenkinson, Mowat, & Mowat, 1992; Fok & Henry, 2015). This could have been another reason why results for optimism did not reach significance, presenting a medium internal consistency rate (acceptable) for the current sample.

Future Brazilian research on aging should endeavor to adapt and validate health measures for the specificities of the targeted elderly population to increase, for instance, the comprehensibility of items and anchors (take a special care of the cultural appropriateness of inverted items), removal of redundant items to make measures more easily applicable and less time-consuming, and compare the validity of the measure against other gold-standard measures or one that directly assesses changes—questioning about perceived changes (Fok & Henry, 2015).

### Strengths and limitations

*Programa Vem Ser* blueprint has shown potential to be useful to health professionals in therapeutic settings for health promotion and disease prevention. PPIs techniques were employed in a structured manner based on CBT perspectives. Some examples of such practices applied in the program are making use of relaxation training and integrating CBT techniques to recognize positive aspects of one’s life/achievements, what one is grateful for, personal strengths/virtues, and how to use those to rise above and grow from adversities. Integrating such factors in intervention designs, and evaluating their results, is of great value to tailor trait-specific PPIs for future use.

Despite the limitations that a non-randomized design may pose, a number of rectifying measures were taken to assure internal validity and warrant relevance of the obtained results. The design included a control group, with multiple sites for data collection, including six collection points at T1 and T2 conducted along the year of 2017. Data collection was conducted simultaneously for the experimental group and controls. This measure accounts for history and maturation effects. Careful consideration of inclusion criteria was also taken to minimize confounding variables between groups.

Similarly, between-group differences at baseline were evaluated prior to data analysis to check for selection bias and comparability/equivalence between conditions. Intention-to-treat analysis (dropout analysis) was also conducted to express the magnitude of generalizability of the results, which indicated equivalence between completers and dropouts, except from depression/anxiety symptoms scores, and perceived social support. These data point out the need to invest in simultaneous practices (constant follow-up, (in) direct contact between sessions, use of technology/gadgets, etc.), perhaps even prior, and/or during the implementation of the program, to increase retention rates of individuals who may present a more at-risk profile at baseline (psychopathology and lacking perceived social support). In other words, our dropout analysis stress the need to offer additional support for those who present higher levels of psychopathology and reduced perceived social support, in an attempt to increase retention as these factors could all be potential barriers to adherence to this sort of health intervention.

Additionally, pre- and post-test evaluations were carried out after a minimum of a 1-month period, and the presentation of the evaluation protocol was counterbalanced to avoid testing and instrumentation effects. All these measures may grant higher credibility to the current findings (Handley, Lyles, McCulloch, & Cattamanchi, 2018). The promising results attained by a multicomponent program format implemented in this context seemed to function well to combine different strengths as a strategy to achieve better health. This was illustrated by the positive results and low attrition rate obtained in this study.

This study has some limitations. The lack of long-term (follow-up) evaluations, to check whether patterns of changes remain constant through time, prevents inferences on the matter of how much variation may be expected/achieved through such intervention in the long run. Similarly, dose analysis including cutoff points to reduce ceiling effects could be useful in future investigations. This would help determine the appropriate length interventions targeting such variables should be to produce advantageous results for this target population. Further investigations are necessary including covariate analyses (socioeconomic status, religious beliefs, sexual orientation, etc.) and subgroup or profile analysis, which are beyond the scope of this paper.

It is also fundamental to include qualitative process evaluation to detect particularly informative elements for clinical practices, such as subjective perception of changes in everyday life and emerging themes of interest that might appear during the implementation process. Those are not always detected by quantitative designs and numerical indicators of health and well-being. Replication studies should ideally include randomization when possible, taking contextual and ethical issues into account, to allow more consistent generalization of these findings beyond the current sample.

## Conclusions

The findings of this study present a range of implications, either for the usefulness or for the applicability of this intervention design for this population group. In sum, the results were satisfactory at T2. It remains questionable however, the extent to which genetic predisposition tends to play a role on the expression of more rigid/inherited traits, depending on individual-specific environmental factors. It could be that longer intervention design is needed for the consolidation of knowledge and buffering effect of such strengths. These could have led to low effects found on measures of empathy and optimism at the end of the intervention. It is also recommended that efforts should be made in prevention–health promotion research to develop or, at last, validate measures for the elderly/retiree populations in different contexts in Brazil. This will reduce measurement error and serve to increase sensitivity to change of the measures.

Though the present findings, it is suggested that the Brazilian public health system should strive to collaboratively broaden the scope of professional training for clinical practices and provide health services towards the development of strengths as protective factors to health. This effort may help achieve treatment goals. Hereto, as beneficial effects were detected in the short-term after the program—to nurture positive affects and cognition and stress-related and internalizing symptoms decline—follow-up studies with the program remain for future investigations. As a final consideration, once intervention programs that incorporate robust designs for the evaluation of effects/impact are yet, in construction in Brazil, more in-depth analyses of *Programa Vem Ser* remain for future studies.

## Appendix

**Table 3:** Session themes, learning objectives, and activities of *Programa Vem Ser*

Themes	Content of session: learning objectives and activities
Session 1: values and self-care/prudence	Rapport building; introduction of the program, moderator, observers and participants; guidelines/norms; opening icebreaking sentence and group debates; psycho-education: values and self-care; group dynamic: panel of values; individual dynamic: health Curtogram form; relaxation training; action plan: Health Protocol
Session 2: optimism	Revision of action plan; opening icebreaking sentence and group debates; psycho-education: optimism; group dynamic1:priming technique; video-debate 1: attributional styles; video-debate 2: problem-solving strategies; individual dynamic: best possible self–positive affirmations; relaxation training; action plan: positive affirmations
Session 3: empathy	Revision of action plan; opening icebreaking sentence and group debates; psycho-education: Empathy; video-debate1:Empathy and Sympathy; group dynamic1:building the Empathy Tree; group dynamic 2:the speech object (self-regulation training); relaxation training; action plan: to be in someone’s shoes
Session 4: gratitude	Revision of action plan; opening icebreaking sentence and group debates; psycho-education: gratitude; video-debate: the ripple; individual dynamic: CV of personal accomplishments; group dynamic: list of creditors; relaxation training; action plan: the gratitude diary
Session 5: forgiveness	Revision of action plan; opening icebreaking sentence and group debates; psycho-education: forgiveness; group dynamic: the surprise balloon (learning from mistakes); individual dynamic: forgiveness letter; relaxation training; action plan: expressive writing
Session 6: meaning of live and work	Revision of action plan; opening icebreaking sentence and group debates; psycho-education: meaning; group dynamic 1: identify strengths; group dynamic 2: identify what is meaningful in life; revision of topics worked along the program; T2 assessment
